# Human Genetic Susceptibility of Leprosy Recurrence

**DOI:** 10.1038/s41598-020-58079-3

**Published:** 2020-01-28

**Authors:** Priscila Verchai Uaska Sartori, Gerson O. Penna, Samira Bührer-Sékula, Maria A. A. Pontes, Heitor S. Gonçalves, Rossilene Cruz, Marcos C. L. Virmond, Ida M. F. Dias-Baptista, Patricia S. Rosa, Maria L. F. Penna, Vinicius Medeiros Fava, Mariane M. A. Stefani, Marcelo Távora Mira

**Affiliations:** 1 Graduate Program in Health Sciences, School of Medicine, Pontifícia Universidade Católica do Paraná, Rua Imaculada Conceição 1155 - Prado Velho, Curitiba Paraná, Brazil; 2 Tropical Medicine Centre, University of Brasília, UnB - Brasília, DF Brazil; 3 Campus Universitário Darcy Ribeiro, S/N, Asa Norte, Brasília - DF, CEP 70.904.970 Brasil; 4 Escola Fiocruz de Governo, Fiocruz Brasília, Avenida L3 Norte, s/n, Campus Universitário Darcy Ribeiro, Gleba A, Brasília Brazil; 5 Tropical Pathology and Public Health Institute, Federal University of Goiás, Rua 235 - s/n - Setor Universitário - Goiania, Goiania, Goiás Brazil; 6 Dona Libânia Dermatology Centre, R. Pedro I, 1033 - Centro, Fortaleza, Ceará Brazil; 7 Tropical Dermatology and Venerology, Fundação Alfredo da Matta, Av. Codajás, 24 - Cachoeirinha, Manaus, Amazonas Brazil; 8 Lauro Souza Lima Institute, Rodovia Comandante João Ribeiro de Barros, km 225/226, s/n - Distrito Industrial Marcus Vinícius Feliz Machado, Bauru, São Paulo Brazil; 9 Epidemiology and Biostatistics Department, Universidade Federal Fluminense, Rua Marquês do Paraná, 303, Centro, Niterói, Rio de Janeiro Brazil; 10 Infectious Diseases and Immunity in Global Health (IDIGH) Program at the Research Institute of the McGill University Health Centre (RI-MUHC), 1001 Boulevard Décarie, Bioinformatics suite ES1.5561, H4A 3J1., Montreal, Quebec Canada

**Keywords:** Molecular medicine, Risk factors

## Abstract

Host genetic susceptibility to leprosy has been intensively investigated over the last decades; however, there are no studies on the role of genetic variants in disease recurrence. A previous initiative identified three recurrent cases of leprosy for which none of the *M. leprae* strains, as obtained in the first and the second diagnosis, had any known genomic variants associated to resistance to Multidrug therapy; in addition, whole genome sequencing indicated that the same *M. leprae* was causing two out of the three recurrences. Thus, these individuals were suspected of being particularly susceptible to *M. leprae* infection, either as relapse or reinfection. To verify this hypothesis, 19 genetic markers distributed across 11 *loci* (14 genes) classically associated with leprosy were genotyped in the recurrent and in three matching non-recurrent leprosy cases. An enrichment of risk alleles was observed in the recurrent cases, suggesting the existence of a particularly high susceptibility genetic profile among leprosy patients predisposing to disease recurrence.

## Introduction

Leprosy is a chronic infectious disease caused by *Mycobacterium leprae*, an obligatory intracellular, slow growing, alcohol-acid resistant bacillus with tropism for skin macrophages and Schwann cells of the peripheral nervous system^1^. The disease affects more than 200.000 people every year globally, with high incidence and prevalence concentrating in India and Brazil^2^. Leprosy presents a large spectrum of clinical manifestations that varies depending on the host’s immune response, ranging from the localized tuberculoid to the systemic lepromatous forms as defined in the classic Ridley & Jopling classification system^3^. Alternative, the World Health Organization (WHO) classification protocol distributes leprosy cases as paucibacillary (PB) or multibacillary (MB) for treatment purposes^4^.

Leprosy is treated by a multidrug therapy (MDT) consisting of rifampicin, dapsone and clofazimine carried out for six monthly doses for PB patients and for 12 monthly doses for MB patients^5^; treatment is distributed for free in endemic countries since 1995^6^. Currently, there is the proposal for a uniform multidrug therapy regimen for leprosy patients that consists of six doses of MDT prescribed regardless of any clinical classification criteria. A Clinical Trial for Uniform Multidrug Therapy Regimen for Leprosy Patients in Brazil, (U-MDT/CT-BR) was recently conducted to compare outcomes of regular 12 doses MDT (R-MDT) and the uniform six doses MDT (U-MDT). The study included results from laboratory tests (bacilloscopic index, serology and histopathology) and clinical evaluation during long term follow-up^7^. Among the recurrent cases of leprosy identified in the U-MDT/CT-BR trial; for three of them, *M. leprae* isolates obtained in the first and second diagnosis were submitted to whole genome sequencing (WGS); analyses did not show any mutations associated with drug resistance. Based on comparative analysis between *M. leprae* genomes obtained in the first and second infection of each patient, the authors concluded for two cases of relapses caused by same strain and one case of reinfection caused by a different *M. leprae* strain^8^.

Considering that (i) all patients were followed and completed the treatment correctly, (ii) U-MDT was efficient in treating the disease, and (iii) no mutation associated with drug resistance was detected in the genomes of any *M. leprae* strain recovered from the three reinvestigated recurrent cases, it is reasonable to assume that leprosy recurrence was not due to characteristics of the etiologic agent but yet, to a combination of continuous exposure to *M. leprae* associated with an increased host susceptibility genetic background.

Host genetics accounts for a great proportion of disease heritability in leprosy and many genes were already identified with substantial evidences in support of their association with leprosy^9^. Among them, genes *HLA-DR-DQ (major histocompatibility complex, class II, DR – class II DQ)*^10^, *LTA* (*lymphotoxin alpha*)^11^, *IL10* (*interleukin 10*)^12^, *PRKN/PACRG* (*parkin RBR E3 ubiquitin protein ligase/parkin coregulated gene*)^13^, *NOD2* (*nucleotide binding oligomerization domain containing 2*)^14^*, LACC1*/*CCDC122* (*laccase domain containing 1/coiled-coil domain containing 122*)^14^, *SOD2* (*superoxide dismutase 2*)^15^, *NEBL* (*nebulette*) (unpublished data), *GATA3* (*GATA binding protein 3*)^16^, *IFNG* (*interferon gamma*)^17^ and *TLR1* (*toll like receptor 1*)^18^ have been associated with leprosy in Brazilian population samples, the majority of them playing important roles related to the host immune system. However, to date, no attempt has been made to translate this genetic knowledge into the definition of an individual susceptibility profile that could help explain complex leprosy phenotypes such as recurrence/reinfection. Here we genotyped 19 Single Nucleotide Polymorphism (SNP) distributed across these 11 *loci* associated with leprosy *per se* on six leprosy cases from the U-MDT/CT-BR cohort: two cases previously reported as relapses and one as reinfection^8^, and three matching, successfully treated leprosy patients that did not relapse. Allele frequencies were compared across the two groups and with publicly available databases.

## Results and Discussion

All 19 SNPs were successfully genotyped for all six samples of leprosy patients and results are summarized in Table 1. A global count including all genes/markers assessed revealed that risk alleles were more frequently observed in recurrent patients #3208 and #2188, in contrast to controls and recurrent patient #1126.

**Table 1:** Genotypes for all markers on all patients.

Gene	Marker	Risk Allele	Frequency on recurrent cases	Frequency on controls	Frequency on public database^a^	Cases			Controls		
						3208	1126*	2188	9162	9638	9536
*IL10*	rs1800871	A	0.67	0.50	0.43	AG	AA	AG	AA	AG	GG
*PARK2/PACRG*	rs2803073	G	0.17	0.33	0.34	AA	AG	AA	AG	AG	AA
	rs1040079	G	0.67	0.0	0.32	GG	AA	GG	AA	AA	AA
	rs2276201	C	0.67	0.0	0.41	CC	TT	CC	TT	TT	TT
	rs9356058	T	0.67	0.17	0.75	TT	CC	TT	CC	CT	CC
*SOD2*	rs4880	G	0.33	0.50	0.41	AG	AA	AG	AG	AA	GG
*NEBL*	rs625903	G	0.33	0.67	0.59	AA	AG	AG	AG	AG	GG
*CCDC122/LACC1*	rs2275252	C	0.17	0.33	0.61	AA	AC	AA	AC	AC	AA
	rs4942254	T	0.17	0.33	0.57	CC	CT	CC	CT	CT	CC
*NOD2*	rs8057341	G	1.00	0.67	0.52	GG	GG	GG	GG	AG	AG
	rs3135499	A	0.17	0.50	0.64	CC	AC	CC	AC	AC	AC
	rs2111234	A	0.83	0.67	0.51	AA	AG	AA	AA	AG	AG
*HLA-DRB1/DQA1*	rs602875	A	0.83	0.67	0.73	AA	AG	AA	AG	AG	AA
	rs1071630	T	0.67	0.50	0.45	TT	CC	TT	CT	CT	CT
*LTA*	rs2239704	A	0.50	0.50	0.35	AA	AC	CC	CC	AA	AC
	rs909253	A	0.67	0.67	0.61	AA	AG	AG	GG	AA	AA
*GATA3*	rs10905284	C	0.67	0.67	0.56	AC	AC	CC	AC	CC	AC
*IFNG*	rs2069727	T	0.83	0.50	0.73	CT	TT	TT	TT	CC	CT
*TLR1*	rs4833095	C	0.83	0.33	0.57	CC	CC	CT	CT	CT	TT

A: adenine, G: guanine, C: cytosine, T: thymine. *Patient suspected of reinfection

^a^: Data from 1000 genomes, phase 3, all superpopulations combined, obtained on Ensembl Genome Browser.

Comparison of the combined genotypic distribution of all variants tested between the two groups revealed an enrichment of homozygous genotypes for the risk alleles among the three recurrent patients (Table 2): 40% vs. 19% among controls (P = 0.02; OR = 2.83; 95% CI 1.22–6.58). The same comparison applied for the combined allelic distribution also revealed an enrichment of risk alleles among cases, with a suggestive difference that failed to reach statistical significance at the 0.05 level (57% vs. 45% among controls; P = 0.08). These results must be interpreted with caution, given the extremely low sample size and the moderate to high degree of linkage disequilibrium across markers located within gene *loci PRKN/PACRG* (*r*^2^ = 1 between rs1040079 and rs2276201 and *r*^2^ = 0.7 between rs1040079 or rs2276201 and rs9356058), *LTA*, (*r*^2^ = 0.5 between rs2239704 and rs909253), *NOD2* (*r*^2^ = 0.6 between rs2111234 and rs8057341, and *r*^2^ = 0.66 between rs2111234 and rs3135499), *HLA-DRB1/DQA1* (*r*^2^ = 0.46 between rs602875 and rs1071630) and *CCDC122/LACC1* (*r*^2^ = 1 between rs2275252 and rs4942254). Importantly: it is possible that high LD is in fact reflecting the additive effect of two or more variants of distinct functional impact upon the net risk for that gene. This effect would be similar to what has been observed previously for gene *TNFSF8*^19^: extensive LD has been detected between associated markers identified as independent expression quantitative trait loci (eQTL) for *TNFSF8*; the authors argue that this combined functional impact could reflect the high correlation observed between the SNP alleles.

**Table 2:** Count of risk allele on cases and controls.

	Cases	Controls	3208	1126*	2188	9162	9638	9536
Risk alleles	65 (0.57)	51 (0.45)	24	18	23	18	18	15
Non-risk allele	49 (0.43)	63 (0.55)	14	20	15	20	20	23
Homozygous for the risk allele	23 (0.40)	11 (0.19)	10	4	9	4	3	4
Non-homozygous for the risk allele	34 (0.60)	46 (0.81)	9	15	10	15	16	15

*Patient suspected of reinfection.

It is interesting to note that recurrent patient #1126 presented allelic and genotypic global counts similar to the controls and in contrast with the two other recurrent individuals. Patient #1126 has been described previously as the only case of recurrence caused by distinct *M. leprae* strains isolated in the first and the second diagnosis^8^. Also, patient #1126 presented the shortest time to relapse and the latest age of diagnosis – 32 years – similar to the controls (Table 3). One cannot disregard the possibility that recurrent patients #3208 and #2188 have also suffered reinfection – and not relapse – caused by the same *M. leprae* strain. This hypothesis is reinforced by the fact that none of the *M. leprae* strains have the classically recognized resistance mutations^8^, thus relapse caused by treatment failure is unlikely. Thus, the most striking host-related, non-genetic difference between recurrent patients #1126 and #3208 and #2188 is the age of diagnosis. In leprosy, the presence of the age-dependent association has already been shown for *PRKN-PACRG*^20^, *LTA*^11^, *miR-146a*^21^ (leprosy *per se*) and *TNFSF15-TNFSF8*^22^ (leprosy reaction). The enrichment of risk alleles observed for recurrent patients #3208 and #2188 is compatible with the hypothesis that early onset leprosy is more heavily dependent on genetic mechanisms, whereas late onset disease is a balanced combination of genetic and environmental factors.

**Table 3:** Demographic and diagnostic characteristics of leprosy patients with/without recurrence.

Patients	Age at First Diagnosis	Ethnic Group	Leprosy Classification	Leprosy Reaction	Time to Relapse (Months)
**Case/Relapse Patients**					
1126*	32	Mixed	BL	T2R/Neuritis/T1R	46
3208	17	Mixed	LL	Neuritis/T1R/T2R	86
2188	20	Mixed	LL	T1R/T2R	79
**Control/Patients Without Relapses**					
9162	35	Mixed	BL	T1R	NA
9536	32	Mixed	LL	Neuritis/T2R	NA
9638	29	Mixed	LL	T2R	NA

Abbreviations: BL: Borderline Leprosy, LL: Lepromatous Leprosy, T1R: Type 1 Reaction, T2R: Type 2 Reaction, ND: not declared, NA: not applicable. *Patient reported as reinfection.

A higher frequency of the risk allele is observed for markers of several well-known leprosy susceptibility genes – *IL10*, *PACRG*, *NOD2*, *HLA-DRB1/DQA1*, *LTA*, *GATA3*, *IFNG* and *TLR1* – when relapse cases are compared with controls and the frequency available on public databases (Table 1). This observation, although interesting, has only qualitative value in the context of a description of a series of cases and should be interpreted with extreme caution.

## Materials and Methods

### Ethics approval

This study was performed following the Declaration of Helsinki and Brazilian research regulations and was approved by the National Ethics Commission of Research (CONEP) of the Ministry of Health, protocol number 12949/2007, as previously described^7^. Informed consent was obtained from all individual participants included in the study.

### Subjects

Six male leprosy patients were selected from the previous U-MDT/CT-BR study; three did not relapse during an eight-year follow-up; two were diagnosed as relapse caused by the same *M. leprae* strain and one was considered a reinfection caused by a distinct *M. leprae* isolate^8^. The three non-recurrent patients were matched with the recurrent cases by leprosy clinical form, ethnicity (estimated based on phenotypical features and skin color and distributed as Caucasian, African, Asian, Native American or Mixed), bacilloscopic index, treatment protocol, occurrence of leprosy reaction and place of residence (all six patients were from a leprosy hyperendemic area of Fortaleza, Brazil). A summary of the demographic and diagnostic information is presented in Table 3.

### Gene selection, genotyping and analysis

Genes were selected for genotypic analysis based on previous evidence for validated/replicated association with leprosy in Brazilian population samples. SNP markers have been selected based on previous published, association with leprosy in one or more Brazilian population samples and/or in previous genome-wide association studies (GWAS) (Supplementary Table S1). Genomic DNA was obtained from peripheral blood by salting-out^23^; the DNA was quantified spectrophotometrically using NanoDrop 2000/2000c and the concentration of genomic DNA was normalized to 20 ng/µL. Genotyping was performed by fluorescence-based allelic discrimination using TaqMan, as implemented in the Applied Biosystems® 7500 Real-Time PCR platform. The reactions mix was composed by ultrapure water (1.9 μl per sample), TaqMan® SNP Genotyping Assays (40×, 0.1 μl per sample), TaqMan™ Genotyping Master Mix (2×, 3 μl per sample) and template DNA (3 μl). Reaction was conducted using a denaturing temperature of 95 °C for 15 seconds and an oligonucleotide hybridization and extension temperature of 60 °C for 2 minutes on each of a total of 50 cycles.

Linkage disequilibrium (LD) was estimated using Haploview v.4.2 using the combined dataset of all six patients. A descriptive analysis was performed using the Statistical Package for the Social Sciences (SPSS) v.22. Genetic profiles of recurrent and non-recurent cases were described against the frequencies of risk/protection alleles obtained from previous studies as available on Ensembl Genome Browser with data of 1000 genomes, phase 3, combined population. The Fisher’s exact test for small sample sizes was used to compare combined allelic and genotypic frequencies across the two groups.

## Conclusion

Our study presents, for the first time, suggestive evidence that genes associated with susceptibility to leprosy may also play an important role on disease recurrence. Our data indicates that a combination of alleles in different genes may confer hyper-susceptibility to leprosy detectable even among leprosy susceptible individuals, increasing the chances of disease recurrence. In addition, one could speculate, based on the allelic profile of patient #1126 – similar to the non-recurrent and distinct of the other two recurrent individuals – on the possible existence of distinct genetic mechanisms controlling disease relapse and reinfection. We are fully aware of the limitations of our design, particularly related to the small sample size; thus, this mainly descriptive, qualitative report of a series of cases must be perceived as a hypothesis-generating initiative, opening the possibility for additional studies of larger sample sizes of non-recurrent and recurrent leprosy affected individuals, aiming to identify biomarkers of risk of reinfection that may be used to monitor treated patients continuously exposed to the bacilli in endemic areas, with potential positive effects over leprosy control programs.

## Supplementary information

Supplementary Dataset Number-Supplementary-information.

## Data Availability

The authors state that all data produced in the study is available.

## References

[1] Britton WJ, Lockwood DN (2004). Leprosy. Lancet.

[2] WHO, W. H. O. Weekly epidemiological record, Global leprosy update, 2017: reducing the disease burden due to leprosy (2018).

[3] Ridley DS, Jopling WH (1966). Classification of leprosy according to immunity. A five-group system. International journal of leprosy and other mycobacterial diseases: official organ of the International Leprosy Association.

[4] World Health Organization. A guide for surveillance of antimicrobial resistance in leprosy: 2017 update. (2017).

[5] WHO/Department of Communicable Disease Prevention, C. a. E. 38 (2000).

[6] WHO. *Multidrug therapy (MDT)*, http://www.who.int/lep/mdt/en/ (2018).

[7] Penna GO (2017). Uniform multidrug therapy for leprosy patients in Brazil (U-MDT/CT-BR): Results of an open label, randomized and controlled clinical trial, among multibacillary patients. PLoS neglected tropical diseases.

[8] Stefani, M. M. A. *et al*. Whole genome sequencing distinguishes between relapse and reinfection in recurrent leprosy cases. *PLoS neglected tropical diseases* **11**, doi:ARTN e000559810.1371/journal.pntd.0005598 (2017). 10.1371/journal.pntd.0005598 PMC5498066 28617800

[9] Sauer ME (2015). Genetics of leprosy: expected and unexpected developments and perspectives. Clinics in dermatology.

[10] da Silva SA (2009). HLA-DR and HLA-DQ alleles in patients from the south of Brazil: markers for leprosy susceptibility and resistance. BMC infectious diseases.

[11] Alcais A (2007). Stepwise replication identifies a low-producing lymphotoxin-alpha allele as a major risk factor for early-onset leprosy. Nature genetics.

[12] Alvarado-Arnez LE (2015). Association of IL10 Polymorphisms and Leprosy: A Meta-Analysis. PloS one.

[13] Mira MT (2004). Susceptibility to leprosy is associated with PARK2 and PACRG. Nature.

[14] Sales-Marques C (2014). NOD2 and CCDC122-LACC1 genes are associated with leprosy susceptibility in Brazilians. Human genetics.

[15] Ramos GB (2016). Association Analysis Suggests SOD2 as a Newly Identified Candidate Gene Associated With Leprosy Susceptibility. The Journal of infectious diseases.

[16] Medeiros P (2016). The GATA3 gene is involved in leprosy susceptibility in Brazilian patients. Infection, genetics and evolution: journal of molecular epidemiology and evolutionary genetics in infectious diseases.

[17] Cardoso CC (2010). IFNG +874T > A single nucleotide polymorphism is associated with leprosy among Brazilians. Human genetics.

[18] Marques Cde S (2013). Toll-like receptor 1 N248S single-nucleotide polymorphism is associated with leprosy risk and regulates immune activation during mycobacterial infection. The Journal of infectious diseases.

[19] Fava VM (2015). Association of TNFSF8 regulatory variants with excessive inflammatory responses but not leprosy per se. The Journal of infectious diseases.

[20] Alter A (2013). Linkage disequilibrium pattern and age-at-diagnosis are critical for replicating genetic associations across ethnic groups in leprosy. Human genetics.

[21] Cezar-de-Mello PF (2014). Pre-miR-146a (rs2910164 G > C) single nucleotide polymorphism is genetically and functionally associated with leprosy. PLoS neglected tropical diseases.

[22] Fava VM, Sales-Marques C, Alcais A, Moraes MO, Schurr E (2017). Age-Dependent Association of TNFSF15/TNFSF8 Variants and Leprosy Type 1 Reaction. Frontiers in immunology.

[23] John SW, Weitzner G, Rozen R, Scriver CR (1991). A rapid procedure for extracting genomic DNA from leukocytes. Nucleic acids research.

