# Supplementary material for: Human Genetic Susceptibility of Leprosy Recurrence
Source: Sci Rep. 2020 Jan 28;10:1284. doi: 10.1038/s41598-020-58079-3 (PMC6987179; doi:10.1038/s41598-020-58079-3)
Supplement: Supplementary file 1 — Supplementary Dataset Number-Supplementary-information. [file 41598_2020_58079_MOESM1_ESM.pdf]

Human genetic susceptibility of leprosy recurrence

Priscila Verchai Uaska Sartori[1]; Gerson O. Penna[2,3]; Samira Bührer-Sékula[4]; Maria A. A. Pontes[5]; Heitor S. Gonçalves[5]; Rossilene Cruz[6] ; Marcos C. L. Virmond[7]; Ida M. F. Dias-Baptista[7]; Patricia S. Rosa[7]; Maria L. F. Penna[8]; Vinicius Medeiros Fava[9]; Mariane M. A. Stefani[4*]; Marcelo Távora Mira[1*]

Supplementary Table S1. Gene functions and respective SNP markers genotyped in the study

Gene	ID (NCBI)	Function (NCBI)	SNP markers
HLA-DRB1/DQA1	3123/3117	Participates in antigen presentation.	rs602875<br>rs1071630
LTA	4049	Involved in mediation of inflammation, immunostimulation, antiviral responses and apoptosis.	rs2239704<br>rs909253
GATA3	2625	Participates in regulation of T-cell development.	rs10905284
IFNG	3458	Mediates cell-based immune response against microbial and viral pathogens.	rs2069727
TLR1	7096	Recognize pathogen-associated molecular patterns (PAMPs) and mediates cytokine production.	rs4833095
IL10	3586	Exerts a pleiotropic effect on immunoregulation and inflammation.	rs1800871
PRKN	5071	Component of the E3 ubiquitin-protein ligase complex, participates in proteasome-mediated protein degradation.	rs2803073
PACRG	135138	*PRKN* co-regulated gene; suppresses cell death induced by accumulation of unfolded Pael receptor, a PRKN substrate.	rs1040079<br>rs2276201<br>rs9356058
SOD2	6648	Participates in mediation of oxidative phosphorylation and oxidative stress control.	rs4880
CCDC122/LACC1	160857/144811	Unknown/Promotes inflammasome and macrophage activation	rs4942254<br>rs2275252
NOD2	64127	Acts recognizing the muramyl dipeptide derived from intracellular bacterial lipopolysaccharides.	rs8057341<br>rs2111234<br>rs3135499
NEBL	10529	Component of focal adhesion complexes.	rs625903
